# Meaningfulness to patents with preclinical AD of thinking measured by digital cognitive tests in Trial Blazer‐Alz III clinical trial

**DOI:** 10.1002/alz70857_103915

**Published:** 2025-12-24

**Authors:** Paul Maruff

**Affiliations:** ^1^ University of Melbourne, Melbourne, VIC, Australia

## Abstract

**Background:**

In clinical trials in preclinical AD, cognition is a main efficacy measure. This is measured using standardized neuropsychological tests. While the cognitive constructs measured by neuropsychological tests are well‐defined, how patients with preclinical AD understand the types of thinking required of these tests remains unknown. This study used a concept elicitation (CE) method to determine how individuals experience the International Shopping List Test (ISLT) Continuous Paired Associate Learning Test (CPAL) and International Digit Symbol Substitution Test‐medicines version (IDSSTm).

**Method:**

Cognitively‐unimpaired (CU) adults (*n*‐30) with abnormal amyloid (AB) levels were recruited from the AIBLcohort (mean age = 71 (SD 13.1), Females = 19, Education = 11yrs (SD 4.2)). Participants were read the standard instructions from each test and then practiced the test. Prior to the real test, participants were instructed that while performing the test, they should try their best, but should simultaneously consider the type of thinking they felt the test measured. Participants were instructed they could say anything they wished and there are no incorrect answers. They were allowed 3‐mins to complete their responses. Test performance scores was not analysed. After each test, the participant was asked, what type of thinking did you have to use when completing that test. The order of administration of tests was pseudo‐randomized between administration sessions. All responses were recorded and then major themes/descriptors identified and tallied.

**Results:**

The most common descriptors used for the ISLT were, memory (97%), remembering (82%), memorizing (72%), recalling (62%), for the CPAL were, learning (88%), remembering (67%), solving (67%), sorting (55%) and for the IDSSTm were matching (81%), pattern matching (79%), organising (63%), problem solving (55%).

**Conclusions:**

Adults with preclinical AD did understand the type of cognition measured by the learning and memory tests (ISLT, CPAL) although the terms used to describe the varied. For the IDSSTm, most adults considered this to be an assessment of matching. Understanding of the how adults with preclinical; AD understand the types o thinking measured by neuropsychological test will help inform models of the clinical meaningfulness of any drug related benefits observed from trials in which they are being used.

